# Prognostic Factors for Contralateral Recurrence of Upper Tract Urothelial Carcinoma after Nephroureterectomy: A Large Multiregional Study

**DOI:** 10.3390/cancers13235935

**Published:** 2021-11-25

**Authors:** Tsu-Ming Chien, Hsiang-Ying Lee, Nirmish Singla, Vitaly Margulis, Yair Lotan, Solomon Lukasz Woldu, Chun-Nung Huang, Ching-Chia Li, Hung-Lung Ke, Wei-Ming Li, Chia-Yang Li, A-Mei Huang, Sheau-Fang Yang, Hung-Pin Tu, Wen-Jeng Wu, Hsin-Chih Yeh

**Affiliations:** 1 Graduate Institute of Clinical Medicine, College of Medicine, Kaohsiung Medical University, Kaohsiung 80708, Taiwan; 1020431@kmuh.org.tw (T.-M.C.); hsiangying@kmu.edu.tw (H.-Y.L.); amhuang@kmu.edu.tw (A.-M.H.); 2 Department of Urology, Kaohsiung Medical University Hospital, Kaohsiung 80756, Taiwan; 1060574@kmuh.org.tw (C.-N.H.); ccli1010@kmu.edu.tw (C.-C.L.); hlke@kmu.edu.tw (H.-L.K.); wmli@kmu.edu.tw (W.-M.L.); wejewu@kmu.edu.tw (W.-J.W.); 3 Department of Urology, School of Medicine, College of Medicine, Kaohsiung Medical University, Kaohsiung 80708, Taiwan; 4 Department of Urology, Kaohsiung Municipal Ta-Tung Hospital, Kaohsiung 80145, Taiwan; 5 Departments of Urology and Oncology, The James Buchanan Brady Urological Institute, Johns Hopkins University School of Medicine, Baltimore, MD 21287, USA; nsingla2@jhmi.edu; 6 Department of Urology, University of Texas Southwestern Medical Center, Dallas, TX 75390, USA; vitaly.margulis@utsouthwestern.edu (V.M.); yair.lotan@utsouthwestern.edu (Y.L.); solomon.woldu@utsouthwestern.edu (S.L.W.); 7 Department of Urology, Ministry of Health and Welfare Pingtung Hospital, Pingtung 90054, Taiwan; 8 Department of Medical Research, Kaohsiung Medical University Hospital, Kaohsiung 80756, Taiwan; chiayangli@kmu.edu.tw; 9 Graduate Institute of Medicine, College of Medicine, Kaohsiung Medical University, Kaohsiung 80708, Taiwan; 10 Department of Biochemistry, School of Medicine, College of Medicine, Kaohsiung Medical University, Kaohsiung 80708, Taiwan; 11 Department of Pathology, Kaohsiung Medical University Hospital, Kaohsiung 80756, Taiwan; sfyang@kmu.edu.tw; 12 Department of Pathology, School of Medicine, College of Medicine, Kaohsiung Medical University, Kaohsiung 80708, Taiwan; 13 Department of Public Health and Environmental Medicine, School of Medicine, College of Medicine, Kaohsiung Medical University, Kaohsiung 80708, Taiwan; p915013@kmu.edu.tw

**Keywords:** contralateral recurrence, nephroureterectomy, chronic kidney disease, white blood cell, inflammation, upper tract urothelial carcinoma

## Abstract

**Simple Summary:**

Recurrence of cancer on the opposite side after the removal of primary upper tract urothelial carcinoma (UTUC) is uncommon, but the risk of subsequent deterioration of kidney function may be severe and result in the need for permanent dialysis. There is a clear correlation between inflammation and tumor development in patients with cancer. As the presence of white blood cells (WBC) in urine is an indicator of local inflammation and a biomarker for bladder recurrence of UTUC, we hypothesized that systemic inflammation is involved in the occurrence of contralateral lesions. We proved that elevated serum WBC, late chronic kidney disease, and multiple tumors are independent prognostic factors for contralateral recurrence. Moreover, in a subgroup analysis, the importance of chronic kidney disease in contralateral recurrence was demonstrated for the first time in a non-Asian population. It is recommended that high-risk patients be closely followed up to monitor the opposite upper urinary tract.

**Abstract:**

This study aimed to examine the prognostic significance of preoperative inflammation-associated blood cell markers in the metachronous contralateral recurrence of upper tract urothelial carcinoma (UTUC). Patients with nonmetastatic UTUC treated in Taiwan and the U.S. between 1990 and 2017 were included. The Kaplan–Meier method was used to calculate the contralateral recurrence rate, and multivariate logistic regression was performed to study the association of blood cell markers and clinicopathological characteristics with contralateral recurrence. Overall, a total of 1039 patients were included in this study, 52 of whom (5.0%) developed metachronous recurrence of the contralateral side. Kaplan–Meier analysis indicated that a history of bladder cancer (*p* = 0.006), multiple tumors (*p* = 0.016), advanced chronic kidney disease (CKD; *p* < 0.001), elevated serum white blood cell (WBC) count (*p* < 0.001), and decreased hemoglobin levels (*p* = 0.001) significantly reduced the contralateral recurrence-free survival. Multivariate analysis showed that multiple tumors (hazard ratio (HR), 1.87; *p* = 0.030), advanced CKD (HR, 2.63; *p* = 0.002) and increased WBC count (HR, 2.60; *p* = 0.001) were independent risk factors for higher contralateral recurrence rate. Notably, advanced CKD was a significant factor regardless of the patient’s region. In summary, multiple tumors, advanced CKD and elevated serum WBC count are independent predictors of contralateral recurrence in patients with UTUC. It is recommended that patients with these adverse characteristics be closely followed up to monitor the opposite upper urinary tract.

## 1. Introduction

Upper tract urothelial carcinoma (UTUC) is a rare cancer that originates from the urothelium along the pelvicalyceal cavities and ureter. UTUC accounts for about 5–10% of all urothelial malignancies, with a male-to-female ratio of approximately 2:1 [1]. However, the incidence of UTUC in Taiwan is as high as 30–40% of all urothelial cancers, and the proportion of women with disease is slightly higher than that of men [2,3]. Due to the multifocal nature of urothelial carcinoma, the standard treatment for invasive, nonmetastatic UTUC is radical nephroureterectomy (RNU) with bladder cuff excision.

Most previous studies have investigated the prognostic factors of cancer progression, intravesical recurrence, and survival outcomes of UTUC; however, few studies have focused on indicators for predicting metachronous contralateral recurrence. The development of contralateral UTUC after removal of the primary lesion is uncommon, with an estimated incidence of 0.6–6.9% [2,4,5,6,7,8,9]. Identifying patients at risk of contralateral recurrence can help early detection to prevent another RNU for recurring contralateral tumors and permanent dialysis.

Inflammation is one of the hallmarks of cancer, and the inflammatory microenvironment is crucial for tumor progression [10,11]. There is a clear correlation between inflammation and survival in patients with malignancies. Pyuria, defined as an elevated white blood cell (WBC) count in the urine, usually indicates an inflammatory process. Preoperative pyuria has been found to be a prognostic biomarker for intravesical recurrence of UTUC [12,13]. As pyuria is an indicator of local inflammation and can predict future recurrence of the bladder, we hypothesized that systemic inflammation is involved in the development of metachronous contralateral recurrence. WBCs and platelets are cellular components of systemic inflammation [11], and hemoglobin is an inflammatory marker associated with tumor-related anemia [14]. All of these markers are readily available in routine blood tests before surgery, but their value in predicting contralateral outcome has never been studied. In addition, there are differences in demographic and clinicopathological characteristics according to the geographical distribution of patients with UTUC [15]. Therefore, we created this multiregional cohort to examine the effects of blood cell markers and other host and tumor features on the contralateral recurrence of UTUC.

## 2. Materials and Methods

### 2.1. Patient Collection

Patients who received RNU for nonmetastatic UTUC at the Kaohsiung Medical University Healthcare System in Taiwan and the University of Texas Southwestern Medical Center in the U.S. from 1990 to 2017 were included. This study was approved by the review board of our institution (KMUHIRB-E(I)-20180214). Before RNU, all patients underwent routine blood tests to obtain WBC, hemoglobin, and platelet values for analysis. Parameters including demographic features, clinicopathological characteristics, oncologic follow-up, and causes of death were collected retrospectively. Patients with unknown WBC count (n = 190) or pathological details (n = 32) were excluded. Tumor stage was determined according to the 2010 AJCC (American Joint Committee on Cancer) TNM system, and tumor grade followed the 2004 World Health Organization/International Society of Urologic Pathology consensus classification. Renal function was assessed by estimated glomerular filtration rate (eGFR), which was calculated using the MDRD (Modification of Diet in Renal Disease) equation [16]. Advanced chronic renal disease (CKD) was defined as an eGFR <30 mL/min/1.73 m^2^.

### 2.2. Postoperative Follow-Up

After RNU, an outpatient clinic was arranged trimonthly in the first 2 years for detailed medical history, physical examination, urinary cytology, and cystoscopy, and every 6 months for the subsequent 2 years, after which, patients with no evidence of disease were followed up annually. Imaging studies such as kidney ultrasound and computed tomography of the abdomen, were conducted regularly in accordance with the surveillance guidelines. Metachronous contralateral recurrence was defined as a histologically confirmed urothelial carcinoma in the opposite upper urinary tract. Adjuvant chemotherapy was administered according to pathological stage, performance status, renal function, and treatment willingness.

### 2.3. Statistical Analysis

Differences between categorical and continuous parameters were evaluated using Pearson’s chi-square test and Student’s *t*-test, respectively. The Kaplan–Meier method was applied to estimate the influence of blood cell markers and clinicopathological factors on contralateral recurrence-free survival. The survival time was recorded from the date of RNU to the date of contralateral recurrence or the date of the last visit. The log-rank test was used to compare survival curves. A multivariate Cox proportional hazards model was employed to determine the independent predictors of contralateral recurrence-free survival. Statistical significance was set at *p* < 0.05. SPSS software version 22 was utilized to perform all statistical analyses.

## 3. Results

The present study included 1039 patients with UTUC, of whom 504 (48.5%) were males and 535 (51.5%) were females. The average age of the patients was 68.1 ± 10.4 years, and the mean follow-up period was 36.0 months. A total of 439 (42.2%), 211 (20.3%), 323 (31.1%), and 66 (6.4%) patients had pTa/pTis/pT1, pT2, pT3, and pT4 tumor stages, respectively. Kaplan–Meier analysis performed using the X-tile program revealed that the optimal cutoff values for WBC, hemoglobin, and platelets were 8.15 × 10^3^ cells/μL, 10.2 g/dL, and 296 × 10^3^ cells/μL, respectively. Table 1 shows that a history of bladder cancer, advanced CKD, increased WBC count, and decreased hemoglobin level are associated with more contralateral recurrences.

### 3.1. Regional Differences

There were 878 (84.5%) Taiwanese patients and 161 (15.5%) American patients. Patients from the U.S. had more males (*p* < 0.001), history of bladder cancer (*p* < 0.001), pelvicalyceal tumors (*p* < 0.001), laparoscopic surgery (*p* = 0.007), early-stage disease (*p* < 0.001), high-grade differentiation (*p* = 0.011), multiple tumors (*p* < 0.001), positive lymph node involvement (*p* < 0.001), adjuvant chemotherapy (*p* = 0.030), and early CKD (*p* < 0.001). Their hemoglobin levels (*p* < 0.001) and platelet counts (*p* = 0.019) were also higher than those of Taiwanese patients. No differences were found in age, smoking, WBC count, or contralateral recurrence (Table 2).

### 3.2. Contralateral Recurrence

**Figure 1:**
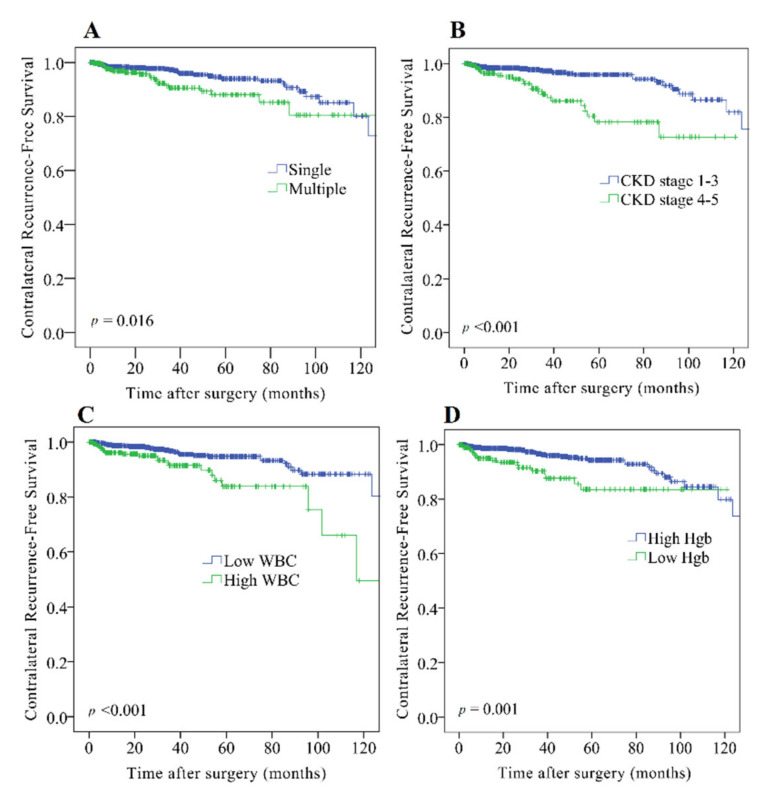
Kaplan–Meier analysis indicated that patients exhibiting (**A**) multiple tumors (log-rank *p* = 0.016), (**B**) advanced chronic kidney disease (CKD) (log-rank *p* < 0.001), (**C**) elevated white blood cell (WBC) count (log-rank *p* < 0.001), and (**D**) decreased hemoglobin level (log-rank *p* = 0.001) had significantly lower contralateral recurrence-free survival rates.

**Figure 2:**
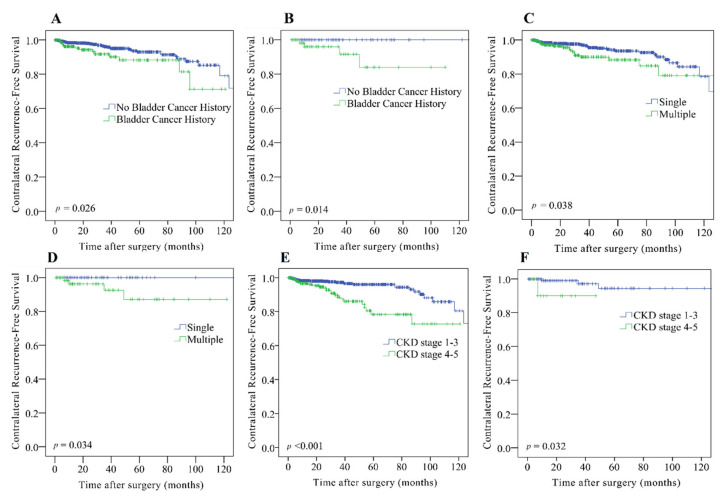
In Kaplan–Meier analysis, bladder cancer history, tumor number, and CKD stage of patients with upper tract urothelial carcinoma (UTUC) from Taiwan (**A**,**C**,**E**) or the U.S. (**B**,**D**,**F**) were significantly associated with contralateral recurrence.

Fifty-two patients (5.0%) from our study cohort had contralateral recurrence, of whom 33 were female and 19 were male. The 3- and 5-year contralateral recurrence-free survival rates were 95.4% and 92.3%, respectively. Kaplan–Meier analysis showed that the contralateral recurrence-free survival rate was significantly reduced in patients with a history of bladder cancer (*p* = 0.006), multiple tumors (Figure 1A, *p* = 0.016), advanced CKD (Figure 1B, *p* < 0.001), elevated WBC count (Figure 1C, *p* < 0.001), and decreased hemoglobin level (Figure 1D, *p* = 0.001). Among these features, bladder cancer history, tumor multifocality, and CKD staging were significant for patients in both Taiwan and the U.S. (Figure 2). Univariate analysis indicated that a history of bladder cancer (*p* = 0.008), multiple tumors (*p* = 0.018), advanced CKD (*p* < 0.001), elevated WBC count (*p* < 0.001), and decreased hemoglobin level (*p* = 0.001) were associated with worse contralateral recurrence-free survival (Table 3). In the multivariate analysis, multiple tumors (hazard ratio (HR), 1.87; 95% confidence interval (CI), 1.06–3.29; *p* = 0.030), advanced CKD (HR, 2.63; 95% CI, 1.42–4.88; *p* = 0.002), and elevated WBC count (HR, 2.60; 95% CI, 1.49–4.54; *p* = 0.001) were independent risk factors for the higher contralateral recurrence rate (Table 3).

It was of concern that the dichotomy of continuous variables may lead to biased estimation, so we tested whether these variables were linearly related to the outcome variable. We found that only hemoglobin had a linear relationship with contralateral recurrence. Therefore, we also used hemoglobin as a continuous variable in the multivariate model, and found that the results were the same as Table 3. Significant factors were still multiple tumors (HR, 1.92; 95% CI, 1.09–3.39; *p* = 0.024), advanced CKD (HR, 2.49; 95% CI, 1.30–4.75; *p* = 0.006), and elevated WBC count (HR, 2.65; 95% CI, 1.51–4.65; *p* = 0.001).

## 4. Discussion

To the best of our knowledge, this is the first study to investigate the importance of inflammation-associated blood cell markers in the contralateral recurrence of primary UTUC after unilateral RNU in a multiregional cohort. The 3- and 5-year contralateral recurrence rates were 4.6% and 7.7%, respectively. We confirmed the finding of previous studies [4,8,9] that advanced CKD is an independent risk factor for contralateral recurrence. Our results also indicated that an elevated WBC count was associated with a higher contralateral recurrence rate. There were no significant differences in overall, cancer-specific, progression-free, and bladder recurrence-free survival rates between patients with and without contralateral recurrence.

Prior or simultaneous presence of bladder cancer has been reported to increase contralateral recurrence [4,5,6,7]. This association partly supports the concept of intraluminal seeding as a mechanism for the development of metachronous contralateral upper urinary tract disease. However, because contralateral retrograde procedures or vesicoureteral reflux are infrequent, previous studies assumed that field cancerization, a theory describing the formation of multiple genetically unrelated tumors caused by carcinogens, may play a dominant role in the growth of contralateral tumors [4,8]. In our results, the importance of multiple tumors in contralateral recurrence supported this idea to some extent. In contrast, a history of bladder cancer was a poor prognostic factor for contralateral recurrence in univariate analysis, but they failed to reach statistical significance in multivariate analysis.

The central hypothesis of this study was that inflammation may promote cancer development at different sites of the urothelium. The WBC count is the most widely used cellular indicator of inflammation and has been repeatedly proven to be a prognostic factor for the survival of patients with UTUC [17,18,19]. The cutoff value ranges from 7.6 × 10^3^ to 8.6 × 10^3^ cells/μL. For the first time, our results showed that a WBC count ≥8.15 × 10^3^ cells/μL was significantly associated with metachronous contralateral UTUC. We believe that cancer-related inflammation can be reflected by an increase in WBC count, leading to the development of contralateral lesions. Decreased hemoglobin level caused by tumor cytokines is an indicator of inflammation [14], but non-cancer-related diseases, such as advanced CKD, can also result in anemia [20]. An increase in platelet count is often a reactive response to chronic inflammatory diseases and cancer [21]. Cytokines released by aggregated platelets can further promote inflammatory reactions and WBC activation [21]. However, an increase in platelet count may be a non-specific response to inflammation [11]. In this study, hemoglobin <10.2 g/dL and platelets ≥296 × 10^3^ cells/μL had a higher tendency of contralateral recurrence in univariate analysis, but lost significance after multivariate adjustment.

Patients with CKD are more likely to develop incident urothelial cancer [22] and have a higher urothelial cancer-specific mortality [23]. CKD is also a risk factor for UTUC [24] and is related to its aggressiveness [25]. In contrast, patients with CKD have no increased risk of prostate, colorectal, lung, or breast cancer [22]. Similar to previous studies [4,8,9], our results indicate that advanced CKD is an independent factor predicting the recurrence of contralateral UTUC after RNU. Of note, because of the lack of CKD data, the significance of CKD in contralateral recurrence has not been studied in non-Asian populations [5,6,7], and our subgroup analysis demonstrated its predictive value in American patients (Figure 2F, *p* = 0.032).

Decreased kidney function causes retention of uremic toxins, which are thought to produce oxidative stress and a chronic inflammatory environment, leading to the activation of the immune response [20,26]. In turn, circulating proinflammatory lymphoid (CD28null T cells) and myeloid (CD16+ monocytes) cell populations expand, but effective immune function decreases [26]. Therefore, the cellular immunity of patients with CKD is impaired. This acquired immunodeficiency can be involved in the occurrence of UTUC and metachronous recurrence of the contralateral side.

It is known that, with the exception of patients in China and Taiwan, men have a higher risk of UTUC than women. Previous studies have shown that women in these areas consume more herbal medicines containing aristolochic acid, resulting in a higher prevalence of UTUC than men [27,28]. The distinct etiology of UTUC in different populations may contribute to the differential impact of gender on UTUC. Female gender was an independent predictor of locally advanced UTUC in French patients [29], but was associated with better survival in Chinese and Taiwanese patients [27,30]. This discrepancy was also observed in the development of contralateral tumors. The contralateral recurrence rates for men and women in Western countries were similar [5,6], but studies in China or Taiwan showed that women had a higher risk of contralateral UTUC than men [2,8,27,28]. In this study, women accounted for 66.7% of Taiwanese patients with contralateral recurrence, compared to only 25.0% of American patients. One reason may be that the proportion of advanced CKD among Taiwanese women and American men was higher than that of their counterparts (American men, 8.8%; American women, 6.3%; Taiwanese men, 18.2%; Taiwanese women, 30.0%). In univariate analysis, women tended to have a higher contralateral recurrence rate (*p* = 0.096).

The occurrence of contralateral recurrence was related to the region. The frequency of contralateral recurrence in Western countries seems to be relatively low: 0.6% in the U.S. [5], 3.1% in Sweden [7], 6.0% in Italy/France [6], 4.5–6.9% in China [8,9], and 4.6–5.8% in Taiwan [2,4]. Except for one article, all analyses in other studies were conducted for hospital patients who were followed up regularly. The analysis from the National Cancer Database showed that the probability of contralateral recurrence is only 0.6% [5], which may be due to coding problems. Although in this study, the incidence of metachronous contralateral UTUC in Taiwanese patients was higher than that in American patients (5.5% vs. 2.5%), geographic area was not a significant factor. The difference in contralateral recurrence rates in different patient regions may also be affected by the incidence of advanced CKD (Taiwan: 24.7%, U.S.: 8.1%).

We proved that whether the patient is in Taiwan or the U.S., advanced CKD is an important risk factor for contralateral recurrence. Nevertheless, when constructing predictive models for different populations, we should carefully consider the background differences in specific variables, such as gender. This study had several limitations. First, this was a retrospective study. Second, the enrolled patients were treated by different surgeons over a 27-year period. Third, only one tertiary medical institution in the U.S. participated in this study. Due to the unique characteristics of Taiwanese patients with UTUC, our results may not be reliably extrapolated to different regions. Fourth, we had no information on other inflammation-based scores or inflammatory markers derived from WBC differential counts to verify the effect of systemic inflammation on metachronous contralateral recurrence. Therefore, it is necessary to conduct large-scale research to validate our findings.

## 5. Conclusions

In summary, multiple tumors, advanced CKD (eGFR < 30 mL/min/1.73 m^2^), and elevated WBC count (≥8.15 × 10^3^ cells/μL) were independent prognostic factors for contralateral recurrence in patients with initial unilateral UTUC receiving RNU. For patients with these three adverse features, close monitoring of the contralateral upper urinary tract during follow-up is recommended.

## Figures and Tables

**Table 1:** Demographics and clinicopathological characteristics of the 1039 patients with upper tract urothelial carcinoma (UTUC) according to contralateral recurrence.

Variables	Total	Contralateral Recurrence		
		Yes	No	*p* Value
	n = 1039	n = 52	n = 987	
Age, years				0.097
Mean ± SD	68.1 ± 10.4	65.6 ± 11.1	68.2 ± 10.4	
Gender				0.076
Male	504 (48.5)	19 (36.5)	485 (49.1)	
Female	535 (51.5)	33 (63.5)	502 (50.9)	
Region				0.111
Taiwan	878 (84.5)	48 (92.3)	830 (84.1)	
U.S.	161 (15.5)	4 (7.7)	157 (15.9)	
Smoking				0.881
No	808 (77.8)	40 (76.9)	768 (77.8)	
Yes	231 (22.2)	12 (23.1)	219 (22.2)	
Bladder cancer history				0.047
No	797 (76.7)	34 (65.4)	763 (77.3)	
Yes	242 (23.3)	18 (34.6)	224 (22.7)	
Tumor location				0.179
Pelvicalyceal Ureteral	484 (46.6) 380 (36.6)	18 (34.6) 22 (42.3)	466 (47.2) 358 (36.3)	
Both	175 (16.8)	12 (23.1)	163 (16.5)	
Type of operation				0.416
Open	443 (42.6)	25 (48.1)	418 (42.4)	
Laparoscopic	596 (57.4)	27 (51.9)	569 (57.6)	
Pathological tumor stage				0.139
pTis/pTa	209 (20.1)	17 (32.7)	192 (19.5)	
pT1	230 (22.1)	11 (21.2)	219 (22.2)	
pT2	211 (20.3)	6 (11.5)	205 (20.8)	
pT3	323 (31.1)	16 (30.8)	307 (31.1)	
pT4	66 (6.4)	2 (3.8)	64 (6.5)	
Nodal status				0.256
N0	298 (28.7)	19 (36.5)	279 (28.3)	
Nx	651 (62.7)	31 (59.6)	620 (62.8)	
N+	90 (8.7)	2 (3.8)	88 (8.9)	
Grade				0.229
Low	159 (15.3)	11 (21.2)	148 (15.0)	
High	880 (84.7)	41 (78.8)	839 (85.0)	
Tumor number				0.120
Single	720 (69.3)	31 (59.6)	689 (69.8)	
Multiple	319 (30.7)	21 (40.4)	298 (30.2)	
Adjuvant chemotherapy				0.716
No	839 (80.8)	43 (82.1)	796 (80.6)	
Yes	200 (19.2)	9 (17.3)	191 (19.4)	
CKD stage				<0.001
Stage 1–3	809 (77.9)	29 (55.8)	780 (79.0)	
Stage 4–5	230 (22.1)	23 (44.2)	207 (21.0)	
WBC ≥ 8.15 × 10^3^ cells/μL				0.019
No	730 (70.3)	29 (55.8)	701 (71.0)	
Yes	309 (29.7)	23 (44.2)	286 (29.0)	
Hgb < 10.2 g/dL				0.012
No	823 (79.2)	34 (65.4)	789 (79.9)	
Yes	216 (20.8)	18 (34.6)	198 (20.1)	
PLT ≥ 296 × 10^3^ cells/μL				0.338
No	851 (81.9)	40 (76.9)	811 (82.2)	
Yes	188 (18.1)	12 (23.1)	176 (17.8)	

SD: standard deviation, UTUC: upper tract urothelial carcinoma, CKD: chronic kidney disease, WBC: white blood cell, Hgb: hemoglobin, PLT: platelet.

**Table 2:** Demographics and clinicopathologic characteristics of the 1039 patients with UTUC according to region of presentation.

Variables	Region		
	Taiwan	U.S.	*p* Value
	n = 878	n = 161	
Age			0.097
Mean ± SD	67.8 ± 10.4	69.3 ± 10.4	
Gender			<0.001
Male	391 (44.5)	113 (70.2)	
Female	487 (55.5)	48 (29.8)	
Smoking			0.232
No	677 (77.1)	131 (81.4)	
Yes	201 (22.9)	30 (18.6)	
Bladder cancer history			<0.001
No	706 (80.4)	91 (56.5)	
Yes	172 (19.6)	70 (43.5)	
Tumor location			<0.001
Pelvicalyceal Ureteral	374 (42.6) 331 (37.7)	110 (68.3) 49 (30.4)	
Both	173 (19.7)	2 (1.2)	
Type of operation			0.007
Open	390 (44.4)	53 (32.9)	
Laparoscopic	488 (55.6)	108 (67.1)	
Pathological tumor stage			<0.001
pTis/pTa	149 (17.0)	60 (37.3)	
pT1	200 (22.8)	30 (18.6)	
pT2	199 (22.7)	12 (7.5)	
pT3	273 (31.1)	50 (31.1)	
pT4	57 (6.5)	9 (5.6)	
Nodal status			<0.001
N0	237 (27.0)	61 (37.9)	
Nx	576 (65.6)	75 (46.6)	
N1	65 (7.4)	25 (15.5)	
Grade			0.011
Low	145 (16.5)	14 (8.7)	
High	733 (83.5)	147 (91.3)	
Tumor number			<0.001
Single	641 (73.0)	79 (49.1)	
Multiple	237 (27.0)	82 (50.9)	
Adjuvant chemotherapy			0.030
No	719 (81.9)	120 (74.5)	
Yes	159 (18.1)	41 (25.5)	
CKD stage			<0.001
Stage 1–3	661 (75.3)	148 (91.9)	
Stage 4–5	217 (24.7)	13 (8.1)	
WBC ≥8.15 × 10^3^ cells/μL			0.834
No	618 (70.4)	112 (69.6)	
Yes	260 (29.6)	49 (30.4)	
Hgb < 10.2 g/dL			<0.001
No	675 (76.9)	148 (91.9)	
Yes	203 (23.1)	13 (8.1)	
PLT ≥ 296 × 10^3^ cells/μL			0.004
No	732 (83.4)	119 (73.9)	
Yes	146 (16.6)	42 (26.1)	
Contralateral recurrence			0.111
No	830 (94.5)	157 (97.5)	
Yes	48 (5.5)	4 (2.5)	

SD: standard deviation, UTUC: upper tract urothelial carcinoma, CKD: chronic kidney disease, WBC: white blood cell, Hgb: hemoglobin, PLT: platelet.

**Table 3:** Univariate and multivariate analyses predicting contralateral recurrence in the 1039 patients with UTUC after nephroureterectomy.

Variables	Contralateral Recurrence-Free Survival			
n = 1039	Univariate	*p*	Multivariate	*p*
	Analysis		Analysis	
	HR (95% CI)		HR (95% CI)	
Age, years	0.99 (0.97–1.02)	0.712		
Gender		0.096		
Male	1			
Female	1.62 (0.92–2.86)			
Region		0.312		
Taiwan	1			
U.S.	1.70 (0.61–4.71)			
Smoking		0.858		
No	1			
Yes	1.06 (0.56–2.02)			
Bladder cancer history		0.008		0.072
No	1		1	
Yes	2.19 (1.22–3.89)		1.71 (0.95–3.06)	
Tumor location		0.058		
Pelvicalyceal	1			
Ureteral	1.56 (0.84–2.91)	0.164		
Both	2.42 (1.16–5.04)	0.018		
Type of operation		0.710		
Open	1			
Laparoscopic	0.90 (0.52–1.56)			
Pathological tumor stage		0.212		
pTis/pTa	1			
pT1	0.55 (0.26–1.17)	0.121		
pT2	0.36 (0.14–0.91)	0.032		
pT3	0.82 (0.42–1.63)	0.576		
pT4	0.84 (0.19–3.67)	0.820		
Nodal status		0.563		
N0	1			
Nx	0.75 (0.42–1.33)	0.325		
N+	0.62 (0.14–2.65)	0.515		
Grade		0.975		
Low	1			
High	1.01 (0.52–1.98)			
Tumor number		0.018		0.030
Single	1		1	
Multiple	1.96 (1.12–3.41)		1.87 (1.06–3.29)	
Adjuvant chemotherapy		0.802		
No	1			
Yes	1.10 (0.53–2.26)			
CKD stage		<0.001		0.002
Stage 1–3	1		1	
Stage 4–5	3.50 (2.01–6.11)		2.63 (1.42–4.88)	
WBC ≥ 8.15 × 10^3^ cells/μL		<0.001		0.001
No	1		1	
Yes	2.72 (1.57–4.73)		2.60 (1.49–4.54)	
Hgb < 10.2 g/dL		0.001		0.088
No	1		1	
Yes	2.66 (1.49–4.73)		1.76 (0.92–3.35)	
PLT ≥ 296 × 10^3^ cells/μL		0.087		
No	1			
Yes	1.76 (0.92–3.36)			

HR: hazard ratio, CI: confidence interval, UTUC: upper tract urothelial carcinoma, CKD: chronic kidney disease, WBC: white blood cell, Hgb: hemoglobin, PLT: platelet.

## Data Availability

The data presented in this study are available upon reasonable request from the corresponding author. The data are not publicly available due to patient privacy.
